# The role of basic psychological needs in the relationships between identity orientations and adolescent mental health: A protocol for a longitudinal study

**DOI:** 10.1371/journal.pone.0296507

**Published:** 2024-01-02

**Authors:** Veljko Jovanović, Aleksandar Tomašević, Dušana Šakan, Milica Lazić, Vesna Gavrilov-Jerković, Marija Zotović-Kostić, Vojana Obradović

**Affiliations:** 1 Department of Psychology, Faculty of Philosophy, University of Novi Sad, Novi Sad, Serbia; 2 Department of Sociology, Faculty of Philosophy, University of Novi Sad, Novi Sad, Serbia; 3 Faculty of Law and Business Studies dr Lazar Vrkatić, Novi Sad, Union University, Belgrade, Serbia; Johns Hopkins School of Medicine and Kennedy Krieger Institute, UNITED STATES

## Abstract

Research into the role of identity orientations (the relative importance an individual places on different personal and social attributes and characteristics when defining her or his identity) in adolescent mental health is extremely limited. Furthermore, the potential mechanisms that might explain the associations between identity orientations and adolescent mental health are poorly understood. This study protocol describes a one-year longitudinal study across three time points to be initiated with the purpose of investigating the mediating role of basic psychological needs satisfaction and frustration in the relationship between identity orientations and various mental health indicators in adolescence. We aim to recruit a large sample of Serbian adolescents (*N* = 2,000 at Time 1), using a two-stage stratified random sampling. The data will be analyzed using the random intercept cross-lagged panel model (RI-CLPM), and the results will be contrasted with the traditional CLPM. The goal of this study is to make a theoretical contribution to research in the fields of identity, self-determination theory, and adolescent mental health, as well as to provide insights towards the development of evidence-based recommendations for creating prevention and promotion programs aimed at improving the well-being of adolescents.

## Introduction

Developmental theories have long pointed out the vital role of the *process* of identity development for adolescent mental health [1, 2], yet it remains largely unknown how and why identity *content* is associated with mental health in adolescence. The dominant theme in identity research throughout the history of the field has been identity processes (how individuals develop their identities), whereas identity content (what identity is, i.e., the attributes which one’s identity is grounded in) has received limited empirical attention [3]. The content of identity can involve a multitude of domains, but the various aspects that comprise the self and identity have typically been divided into two components: one reflecting individual or personal attributes and characteristics, and the other involving social relationships and collective bonds [4]. Therefore, identity content encompasses a broad range of personal and social self-attributes through which individuals define themselves in relation to other people and the sociocultural context they live in.

The distinction between personal (independent) and social (interdependent) aspects of the self and dimensions of self-construal is at the core of several prominent models of self and self-concept. For example, Markus and Kitayama [5] famously distinguished between independent and interdependent selves (i.e., construals of the self), whereas Sedikides and Brewer [6] posited that the complexity of self-concept is best captured by differentiating three levels of self-representation: the individual self, the relational self, and the collective self. The differentiation of the social dimensions of self-construal was also emphasized by Cross and colleagues [7] who elaborated on the differences between relational-interdependent and collective-interdependent (i.e., group-centered) self-construal. Two central dimensions of the structure of identity (personal and social) [8] have been further refined over the past few decades in an effort to address the variety of attributes composing construals of the self and to enable a better understanding of the role of self-attributes in individuals’ behavior, motivation, and well-being. One of the models that was developed to provide a more comprehensive approach to the structure of identity is the *tetrapartite model of the self* [9], the theoretical framework underlying the present study.

The principal concept of the tetrapartite model of the self has been termed *identity orientations*, which encompasses individual differences in the relative importance an individual places on different personal and social self-attributes. The model makes a distinction between four identity orientations: personal, relational, collective, and public. Individuals with *personal identity orientation* value attributes and traits that make a person feel unique and distinct from other individuals, and this identity orientation is closely associated with independent self-construal [5, 10]. *Relational identity orientation* (valuing attributes that include relationships with close people, such as with romantic partners or close friends), *public identity orientation* (valuing attributes that include one’s reputation, popularity, physical appearance, and public image), and *collective identity orientation* (valuing attributes that include relationships with groups, the community, and collectives) capture three related, yet distinct social, interpersonal aspects of the self, closely associated with interdependent self-construal [11]. The key distinguishing advantages of the tetrapartite model of the self are the inclusion of *public identity orientation* as a fourth fundamental aspect of the self and the recognition of the distinction between the relational and collective aspects of the self, which enables a more comprehensive approach to the structure of identity. In order to assess these four aspects of the self, Cheek and colleagues have developed the Aspects of Identity Questionnaire (AIQ-IV), which measures individual differences in the subjective value people place on different dimensions of the self (for a review of the history of the development of the AIQ-IV, see [9]).

The tetrapartite model of the self has rarely been tested in the field of adolescent mental health and well-being [12], with the limited findings suggesting that different identity orientations might have both positive and negative effects on mental health, while the valuing of public aspects of identity may be most detrimental for well-being [9]. One of the main limitations of this model is an insufficient understanding of the factors that might explain the varying effects of identity orientations on mental health. The present study aims to advance the tetrapartite model of the self by introducing the basic psychological needs [13] as factors that could potentially contribute to understanding how identity orientations and mental health might be linked in adolescence. It is important to note that no evidence was found of the relations between identity orientations and basic psychological needs having been previously investigated. Therefore, we decided to take a more exploratory approach to the study of the role of basic psychological needs in the relationship between adolescent identity orientations and mental health, though certain fundamental hypotheses have been formulated based on both theories, as described below.

The *basic psychological needs theory* [14] specifies three fundamental psychological needs: *autonomy* (acting with a full sense of willingness, authenticity, and volition), *competence* (experience of effectiveness and capability to achieve desired goals), and *relatedness* (feelings of intimacy, connection with, caring for, and being cared for by other people and social groups). Recent research has introduced the dual-process model of basic psychological needs, making a distinction between two separate yet related dimensions: need satisfaction and need frustration [15–17]. The satisfaction of these three needs is considered a fundamental precondition for human well-being and adjustment, whereas the frustration of these needs is assumed to lead to maladaptive behavior and mental health problems. Assumptions of the relative independence of the experiences of need satisfaction and need frustration and the significance of psychological need frustration in ill-being beyond low need satisfaction have received substantial empirical support [17].

As these three universal needs are inherent parts of any human action and fundamental building blocks of human well-being [18], it is theoretically justified to hypothesize that the satisfaction and frustration of the needs for autonomy, competence, and relatedness might serve as mechanisms linking identity orientations and adolescent mental health. Given that identity orientations are not evaluative (i.e., they refer to the centrality of various attributes to one’s sense of identity, but they do not capture whether an individual’s needs in a certain domain important for identity are satisfied), we postulated that beneficial or detrimental effects of identity orientations on adolescent mental health might be mediated through basic psychological needs satisfaction or frustration. Identity orientations accommodate both the personal and social aspects of the self and might motivate people to resolve these three fundamental human needs. In other words, they might serve as a motivational force by stimulating individuals to pursue experiences congruent with valued attributes. For example, individuals with high personal identity orientation could be reasonably expected to engage in activities fostering their competence and autonomy, whereas individuals with high relational identity orientation (as well as those with high collective identity orientation) could be logically expected to seek out activities in which they would presume to experience connection and fulfillment of their need to belong, which, if realized, would in turn likely lead to greater well-being.

In this study, both positive (life satisfaction and positive affect) and negative, i.e., ill-being indicators (depression, anxiety, negative affect, and externalizing behaviors) of mental health are to be included in order to test whether the satisfaction and frustration of basic psychological needs play different roles in adolescent well-being and ill-being, as could be expected by the dual-process model of basic psychological needs. For example, the frustrations of basic psychological needs could be expected to have stronger mediating effects than the low satisfaction of needs in the relationship between identity orientations and ill-being indicators.

### Objectives

**Fig 1:**
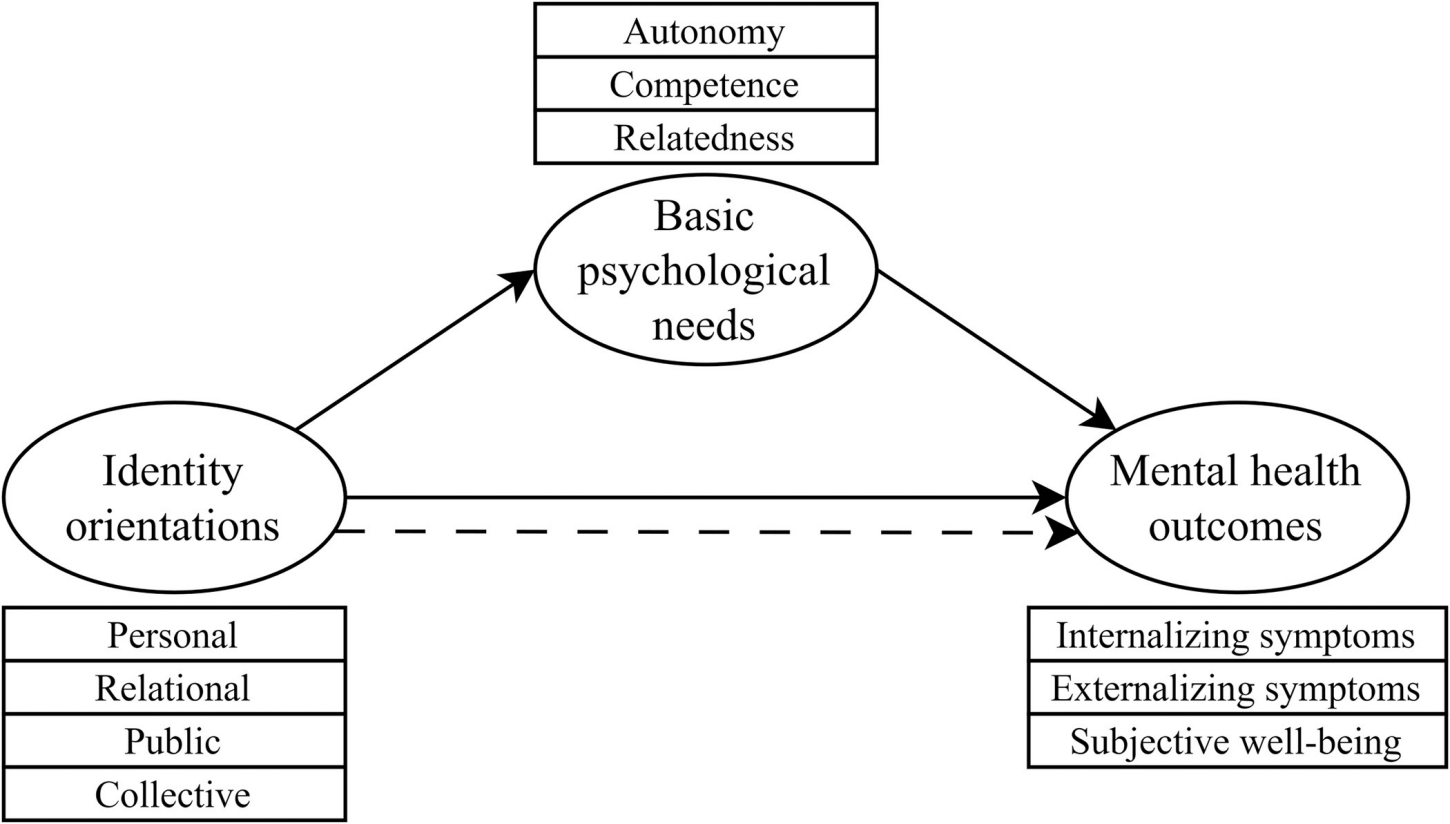
Graphical representation of the mediation model. Control variables (gender, self-rated family’s financial situation, parental employment status, and parental educational level) are not shown for greater clarity.

The main objectives of this longitudinal study are twofold:

1. to investigate the concurrent and longitudinal associations between four identity orientations (i.e., personal, relational, collective, and public) and a range of mental health indicators (externalizing symptoms, depression, anxiety, negative affect, positive affect, and life satisfaction) among adolescents;
2. to examine the mediating roles of the satisfaction and frustration of basic psychological needs (autonomy, competence, relatedness) in the relationship between identity orientations and mental health (see Fig 1 for a graphical representation of the mediation model).

## Materials and methods

This study will employ a longitudinal design, i.e., a three-wave cross-lagged panel design over a one year period. To be examined in the study are the relationships between identity orientations, mental health outcomes, and basic psychological needs satisfaction and frustration on a large sample of Serbian adolescents, who will be mostly aged 15–16 years at the Time 1 assessment. The data, materials, and code are to be made available on the Open Science Framework (OSF). The study was approved by the Ethics Committee of the Faculty of Philosophy, University of Novi Sad (Approval Code: 02-313/4-1).

### Participants and procedure

A large sample of approximately 2,000 secondary school students in Serbia will be recruited and involved as participants at the initial Time 1 assessment. At that time, all participants will be second-year students in high school, largely aged 15–16 years. The selection of the participants shall be carried out through two-stage stratified random sampling, where a sample of schools is to be drawn as a first stage and two or three classes (depending on the class size) then selected from each of the sampled schools as a second stage. The secondary schools will be stratified according to the following features: regional location (four strata: Vojvodina, Belgrade, Šumadija and Western Serbia, Southern and Eastern Serbia), type of settlement (two strata: cities and towns), and type of school (two strata: general schools and vocational schools). It is envisaged that schools that decline participation shall be replaced with the next school featuring the same characteristics, using the list of all secondary schools in Serbia. Self-report questionnaires will be distributed and administered using computer-based (the Jotform Mobile Forms application on tablet devices) and standard paper-and-pencil administration modes. The survey will be administered in a group setting, during regular school classes. In line with recent best-practice recommendations for matching participants across waves in longitudinal studies [19], self-generated identification codes will be employed, in order to ensure confidentiality and anonymity. Participation in the study is to be voluntary and only adolescents who give informed consent will be included.

### Assessment plan

The data will be collected at three time points, spaced approximately six months apart. Time 1 (T1; baseline), Time 2 (T2), and Time 3 (T3) assessments will be conducted in September/October 2023, March/April 2024, and September/October 2024, respectively. At each time point, all measures described below will be administered. Serbian versions of the questionnaires will be used.

### Instruments

#### Independent variables

Identity orientations will be measured using the Aspects of Identity Questionnaire (AIQ-IV; [9]). The original AIQ-IV includes a total of 45 items, but ten items are Special Items which are not scored. Therefore, for the purposes of this study only those 35 items which are scored will be included (such an application has been approved by Jonathan Cheek, one of the authors of the scale). In the questionnaire, each item describes a different aspect of identity, and participants are asked to report how it applies to them on a 5-point scale, from 1 (*Not important to my sense of who I am*) to 5 (*Extremely important to my sense of who I am*). The AIQ-IV was designed to measure four identity orientations: personal (10 items; e.g., *My personal goals and hopes for the future*), relational (10 items; e.g., *Having mutually satisfying personal relationships*), public or social (7 items; e.g., *My attractiveness to other people*), and collective (8 items; e.g., *My feeling of pride in my country*, *being proud to be a citizen*). Three items have been slightly reworded to be more suitable for Serbian adolescents: (a) “My race or ethnic background” has been changed to “My ethnic or national background,” for race has been deemed a relatively irrelevant construct in the Serbian context; (b) “My religion” has been changed to “My religion or my views on religion,” due to not all adolescents being religious; and (c) “My commitments on political issues or my political activities” has been changed to “My opinions on political issues and social problems (such as poverty, unemployment, etc.),” assuming that most adolescents are relatively not politically active.

#### Mediators

Basic psychological needs satisfaction and frustration will be assessed using the Basic Psychological Need Satisfaction and Frustration Scale (BPNSFS; [20]). BPNSFS is a 24-item measure of autonomy, relatedness, and competence satisfaction and frustration. Each of the six dimensions is assessed with four items (e.g., Autonomy satisfaction: *I feel that my decisions reflect what I really want*; Autonomy frustration: *I feel pressured to do too many things*; Relatedness satisfaction: *I feel connected with people who care for me*, *and for whom I care*; Relatedness frustration: *I have the impression that people I spend time with dislike me*; Competence satisfaction: *I feel competent to achieve my goals*; and Competence frustration: *I feel disappointed with many of my performances*). Items are rated on a 5-point scale, from 1 (*not true at all*) to 5 (*completely true*).

#### Outcomes

Internalizing problems (depression and anxiety symptoms) will be assessed using the Depression and Anxiety Scales of Depression Anxiety and Stress Scales 21 (DASS-21; [21]). Each scale consists of 7 items (sample Depression item: *I felt I wasn’t worth much as a person*; sample Anxiety item: *I felt I was close to panic*), rated on a 4-point scale (from 0 = *did not apply to me at all* to 3 = *applied to me very much*, *or most of the time*).

Externalizing problems will be measured using the Youth Externalizing Problems Screener (YEPS; [22]). YEPS is a 10-item measure of externalizing behaviors, covering symptoms of hyperactivity–impulsivity/inattention (e.g., *I get distracted by the little things happening around me*) and conduct problems/oppositional defiance (e.g., *I fight and argue with other people*). Items are rated on a 4-point scale that ranges from 1 (*almost never*) to 4 (*almost always*).

Positive and negative affect will be measured using the Scale of Positive and Negative Experience (SPANE; [23]). SPANE is a 12-item measure of positive (items: positive, good, pleasant, happy, joyful, and contented) and negative (items: negative, bad, unpleasant, sad, afraid, and angry) emotional experiences experienced in the four weeks prior to the time of testing. Items are rated on a 5-point scale (from 1 = *very rarely or never* to 5 = *very often or always*).

Finally, life satisfaction will be measured using the Satisfaction with Life Scale—3 item version (SWLS-3; [24]). SWLS-3 is an abbreviated version of the original SWLS [25], wherein only the first three items are used (i.e., *In most ways my life is close to my ideal*; *The conditions of my life are excellent*; and *I am satisfied with my life*). Items are rated on a 7-point scale that ranges from 1 (*Strongly disagree*) to 7 (*Strongly agree*).

#### Control variables

The following sociodemographic variables will be utilized as control variables: gender (male, female), self-rated family’s financial situation (from 1 = *poor* to 5 = *excellent*), parental employment status (employed, unemployed, retired), and parental educational level, i.e., the highest level of school completed by the mother/father (no school or no elementary school completed, elementary school, secondary school, incomplete university degree, university degree).

### Data analysis strategy

#### Preliminary analyses

Prior to the main analyses, the following preliminary analyses shall be performed: attrition analyses, testing the factor structure, and longitudinal measurement invariance of the measures.

The **attrition analyses** will be conducted using logistic regression [26]. The relations between drop-out in T2 and T3 and study variables assessed at the previous time point will be examined. Drop-out will be coded = 1 and retention = 0, so that odds ratios (ORs) larger than 1 indicate a greater likelihood of drop-out.

**The underlying factor structure** of adolescents’ responses to items measuring constructs of interest in this study will be examined prior to the measurement invariance testing. Due to the complexity of the models applied in this study, measurement models will be evaluated for AIQ-IV, BPNSFS, DASS-21 Depression and Anxiety Scales, YEPS, and subjective well-being (SPANE and SWLS-3 (The single-factor CFA model with three indicators is just-identified, so we aim to evaluate the fit of the three-factor model of subjective well-being as measured by the SWLS-3 and SPANE.)), separately at each of the three time points. The factor structure of the AIQ-IV has rarely been investigated, but it is expected that the four-factor oblique CFA model will provide the best representation of the data. Recent findings suggest that the structure of the BPNSFS is best represented by the bifactor-ESEM model [27], so the applicability of this model to the data of the study will be investigated. More specifically, we aim to contrast CFA, bifactor-CFA, ESEM (Exploratory Structural Equation Modeling), and bifactor-ESEM models following the procedure recommended by Morin et al. ([28]; for details see also [29]). The same procedure will be applied in testing the factor structure of the responses to the DASS-21 Depression and Anxiety scales, in order to evaluate whether any meaningful G-factor of emotional distress and specific factors of Depression and Anxiety [30] can be identified and used in subsequent analyses. For YEPS [22] a good fit is expected in regard to the one-factor CFA model, whereas for subjective well-being (SWLS-3 and SPANE [31]) it is expected that both three-factor oblique CFA and ESEM models will be shown to be adequate.

Models will be estimated in Mplus 8.9 [32] using the Full Information Maximum Likelihood (FIML) procedure to handle the missing data. As most of the indicators can be treated as ordered categorical, the Weighted Least Squares Means and Variance Adjusted (WLSMV) (The robustness of the findings shall also be verified through the application of the Robust Maximum Likelihood (MLR) estimator, which is appropriate when indicators are treated as continuous.) estimator will be employed. The following fit indices will be reported: the Comparative Fit Index (CFI), the Tucker-Lewis Index (TLI), and the Root Mean Square Error Approximation (RMSEA) with 90% confidence interval (CI). Models will be considered good if CFI and TLI values are above .95, and the upper limit of a 90% CI for the RMSEA values are below .06, whereas the thresholds for an acceptable fit to the data will be .90 for the CFI and TLI, and .08 for the RMSEA [33].

Using the final retained measurement models, testing for measurement invariance across time shall then proceed.

The **longitudinal measurement invariance** of the measurement models will be tested in order to evaluate whether measures have the same measurement properties across the three time points [34]. The following two invariance models (i.e., levels of invariance) will be evaluated: (1) the configural invariance model (no equality constraints on the parameters are imposed) to examine whether the same factorial structure holds across time; and (2) the scalar invariance model with both factor loadings and thresholds constrained to be equal across time. For categorical data, it is envisaged and recommended that the factor loadings and thresholds be constrained in tandem, alongside the omission of the separate step of metric invariance which is included when data are treated as continuous [35]. Models will be compared using the changes in CFI and RMSEA values [36]. A drop of .01 in the CFI and an increase of .015 in the RMSEA of the more restricted model compared to the preceding model shall signify a substantial deterioration in model fit and a lack of measurement invariance. In the case of full measurement invariance being rejected, partial invariance will be tested for by freely estimating factor loadings and thresholds in tandem, following the procedure for ordered categorical data [32]. In line with Dimitrov’s [37] recommendations, evidence of and support for partial invariance will be recognized if the proportion of noninvariant parameters to all parameters is less than 20%.

Factor scores from the most invariant models will be saved and used in the main analysis–Random Intercept Cross-Lagged Panel Model (RI-CLPM; [38])–to enable the partial control of measurement errors.

#### Main analyses—Random intercept cross-lagged panel model (RI-CLPM)

RI-CLPM has been proposed as an extension and an alternative to the widely used CLPM approach (also typically referred to as the autoregressive cross-lagged model), which conflates between-person and within-person associations, thus providing inaccurate estimates of reciprocal relations [39]. In contrast to the standard CLPM, the RI-CLPM model estimates both between-person differences in a hypothetically perfectly stable trait (i.e., random intercepts) and another component reflecting state-like (i.e., temporal) deviations from the perfectly stable trait component. Autoregressive paths in RI-CLPM reflect the amount of within-person carry-over effect, i.e., the extent to which temporary deviations are correlated over time. For example, a positive autoregressive path indicates that individuals who score above (or below) their latent trait mean at one time point will also score, at the next time point, above (or below) their latent trait mean. Cross-lagged paths in RI-CLPM capture the longitudinal effects of the time-specific changes on one variable (i.e., within-person deviations from the trait level) on the time-specific increase or decrease on another variable at a later time point. Due to the complexity of this study’s data, the RI-CLPM models will be specified based on the results of previous analyses (i.e., the retained models), and separate RI-CLPMs will be performed for each outcome measure.

In line with recent applications of the RI-CLPM for investigating mediating effects [40], the following models will be tested: Model 1 –direct associations between identity orientations (predictors) and mental health indicators (outcomes); Model 2 (full mediation model)–direct associations between predictors and basic psychological needs satisfaction and frustration (mediators), and between mediators and outcomes; and Model 3 (partial mediation model)–direct associations between predictors and mediators, between mediators and outcomes, and between predictors and outcomes. All associations (autoregressive, cross-lagged, and time-specific) and latent means will be constrained to be equal over time in Models 1–3. After the best fitting model is obtained, the study shall proceed to the evaluation of four alternative models to examine whether latent means (Model 4), autoregressive associations (Model 5), cross-lagged associations (Model 6), and time-specific correlations (Model 7) should be freely estimated. The robustness of the results will be checked by controlling for the following sociodemographic variables: gender, parental employment status, parental educational level, and self-rated family’s financial situation.

**Fig 2:**
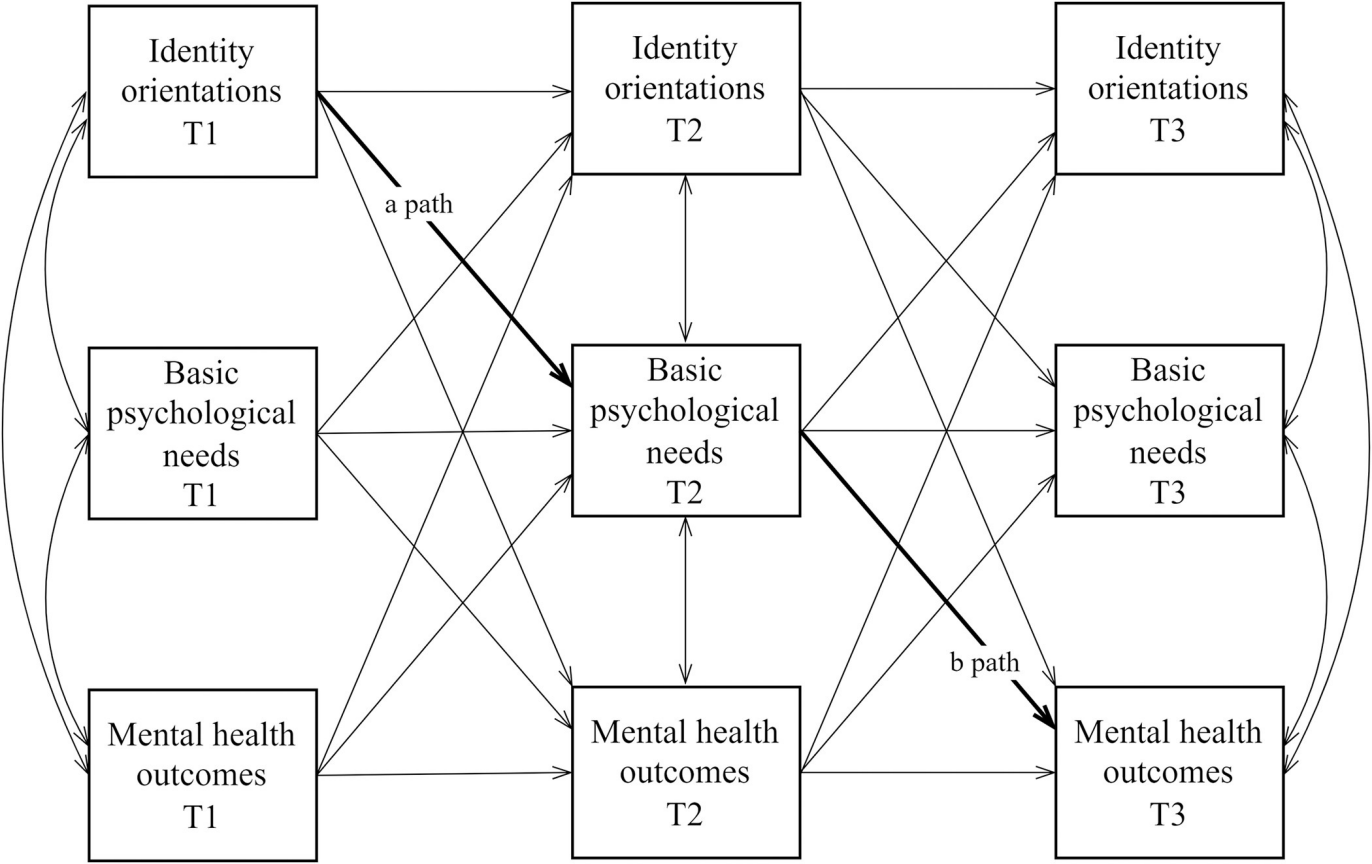
Simplified graphical representation of the cross-lagged panel model. Control variables (gender, self-rated family’s financial situation, parental employment status, and parental educational level) and arrows for correlations between identity orientations and mental health outcomes at Time 2 are not shown, for the sake of clarity. T1 = Time 1, T2 = Time 2, T3 = Time 3.

**Fig 3:**
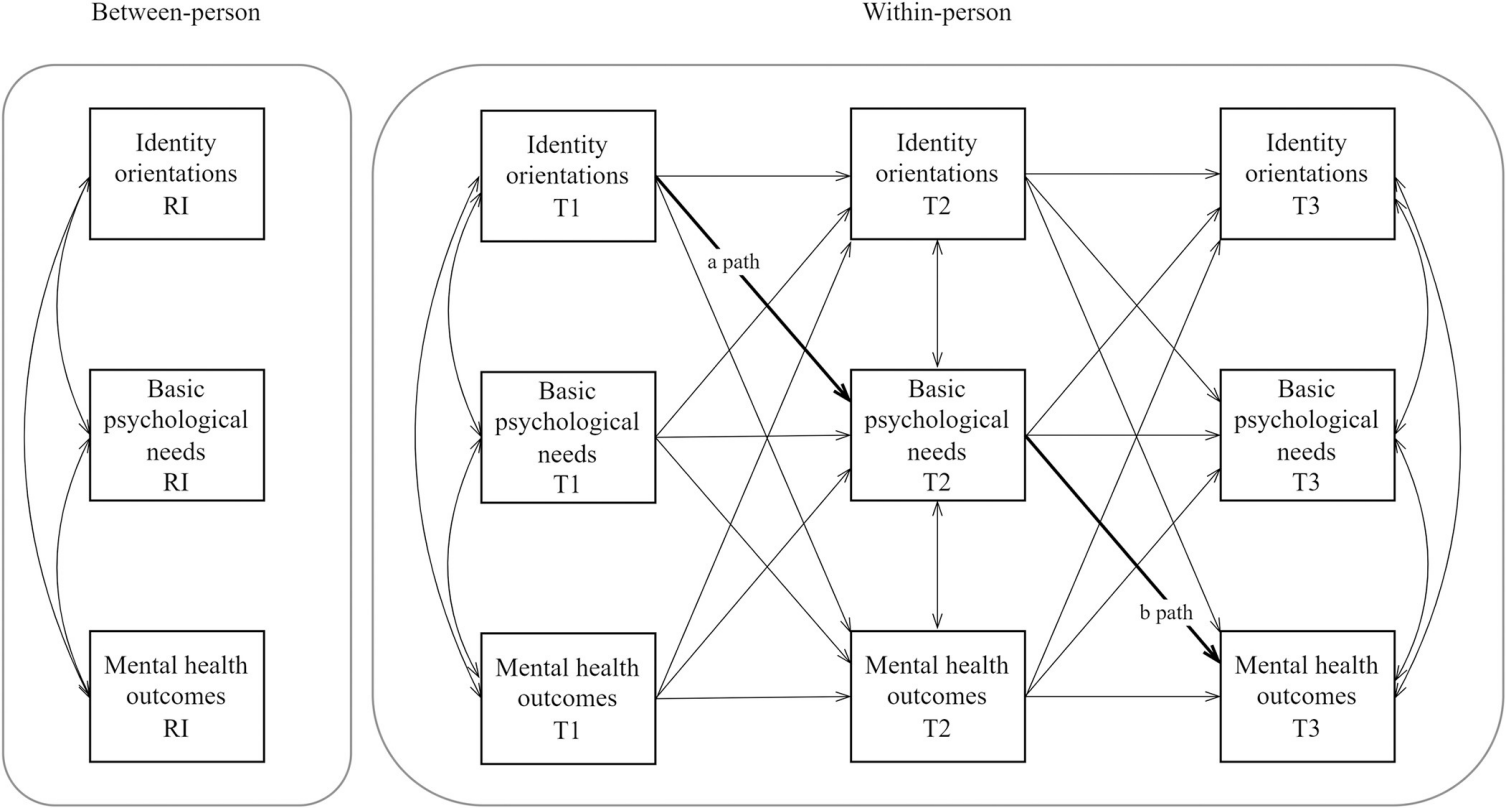
Simplified graphical representation of the random-intercept cross-lagged panel model. Control variables (gender, self-rated family’s financial situation, parental employment status, and parental educational level) and arrows for correlations between identity orientations and mental health outcomes at Time 2 are not shown, for the sake of clarity. RI = Random intercept, T1 = Time 1, T2 = Time 2, T3 = Time 3.

Given the still ongoing debate regarding the pros and cons of the traditional CLPM and the RI-CLPM (for detailed discussion see [39, 41, 42]), the CLPM will also be applied in order to compare these results with those obtained from the RI-CLPM (see Figs 2 and 3 for graphical representations of the CLPM and RI-CLPM models, respectively).

## Discussion

This protocol describes a one-year longitudinal study which aims to deepen and broaden our understanding of the relationships between identity orientations (e.g., the centrality of personal aspects and different social aspects of the self in one’s identity), basic psychological needs satisfaction and frustration, and adolescent mental health. It is intended that the tetrapartite model of the self [9] and the basic psychological needs theory [14] be integrated as frameworks for understanding mental health outcomes in adolescence.

Despite its apparent promise as a more comprehensive model of identity content and self-construal, the tetrapartite model of the self has received limited attention in adolescent mental health research. Furthermore, the potential mechanisms linking identity orientations and mental health in adolescents are still unclear. The proposition of this study is that identity orientations might constitute an important factor for explaining mental health in adolescence, and the basic psychological needs of autonomy, relatedness, and competence satisfaction and frustration are proposed as potential mechanisms for explaining the beneficial or detrimental role of identity orientations on adolescent mental health. The main goal of the study is to investigate whether the satisfaction and frustration of basic psychological needs could be a process through which identity orientations act to improve or impair mental health in adolescence. One of the key postulates of the self-determination theory [14] is that choosing goals and actions that are consistent with one’s self is necessary to achieve optimal functioning. Accordingly, greater efforts are made when one’s self and personal goals are in alignment, which increases the possibility that needs will be met. In addition, according to the self-concordance model [43], individuals must have a sense of who they are and what they value in order to make decisions that are self-congruent and thus satisfy their basic needs. Therefore, it is hypothesized in this study that identity orientations will predict need satisfaction, which in turn will predict mental health outcomes.

A three-wave longitudinal design will enable a more rigorous investigation of the mediation effects of basic psychological needs satisfaction and frustration than that of the cross-sectional design typically used in psychology research. Furthermore, the longitudinal data that the study is intended to generate would allow for the testing of a number of theoretically justified alternative mediation models [44]. One such example that could be tested for its tenability in this context is the reverse mediation model, in which identity orientations would serve as mediators in the relationship between basic psychological needs and mental health outcomes. Basic psychological needs satisfaction can be expected to promote a healthy identity development and a stable sense of self [45, 46], which in turn might lead to better mental health. The focus of this study is to examine the mediating roles of basic psychological needs satisfaction and frustration in the relationship between identity orientations and adolescent mental health. Therefore, it is intended that alternative mediation models shall not be evaluated. In this light, however, we invite researchers to take advantage of the materials and datasets to be made publicly available to develop and test additional meaningful models on the interplay between identity orientations, basic psychological needs, and mental health.

There are several limitations of this study, primarily due to limited study resources. First, the intended sample will include only students in the second year of high school. Therefore, it will not investigate the proposed model across different phases of adolescence (i.e., early, middle, and late). Second, mental health indicators will cover only externalizing and internalizing symptoms and subjective well-being. The authors acknowledge that a comprehensive assessment of adolescent mental health should include a much wider range of indicators, such as prosocial and antisocial behaviors, interpersonal and relational well-being, and psychological well-being (e.g., meaning of life), among other relevant outcomes. Third, the study is reliant on self-report measures, which are essential for the assessment of psychological content of identity, basic psychological needs, and well-being, but have some innate limitations. Finally, the study will cover only a one-year period, which is too brief to uncover longer developmental trends in our data.

Despite these limitations, this study still has the potential to make a notable theoretical contribution to research in the fields of identity, self-determination theory, and adolescent mental health and well-being. In addition, the anticipated impact of the study is that it shall further inform the development of evidence-based guidelines and recommendations for creating prevention and promotion programs aimed at fostering healthy adolescent development.
